# A Decade of Lessons Learned from Integration Strategies in the Netherlands

**DOI:** 10.5334/ijic.5703

**Published:** 2021-11-08

**Authors:** Henk Nies, Diny Stekelenburg, Mirella Minkman, Robbert Huijsman

**Affiliations:** 1 Director of Strategy & Development, Vilans, Centre of Expertise for Long-term Care, Utrecht, The Netherlands; 2 Professor of Organization, Policy Development in Long-term Care, Vrije Universiteit, Amsterdam, The Netherlands; 3 Researcher, Vilans, Centre of Expertise for Long-term Care, Utrecht, The Netherlands; 4 CEO, Vilans, Centre for Expertise for Long-term Care, Utrecht, The Netherlands; 5 Professor of Innovation of organization and governance of integrated long term care, University of Tilburg, The Netherlands; 6 Professor of Management and Organization of Care for Older People, Erasmus University, Rotterdam, The Netherlands; 7 CEO Geriant, Organization for diagnostics, treatment and case management for people with dementia, The Netherlands

**Keywords:** system integration, integration strategies, governance, policy measures, Netherlands

## Abstract

**Introduction:**

In the Netherlands multiple single, cross sector and cross governance level policy reforms were introduced to improve health and social care and decrease fragmentation. In addition to legislative and funding measures, the governmental strategy was to set up long-lasting improvement programs and supported by applied research.

**Description:**

Five national improvement programs on chronic disease management, maternity care, youth care, care for older people and dementia care were analysed. The Laws of integration of Leutz were used as an analytical framework. The programs demonstrated a mixture of employing policy, quality and financial measures to stimulate coherence and integration.

**Discussion:**

The Laws that Leutz formulated are to a large extent applicable in the Dutch context. However, the characteristics of the system of governance being corporatist in its structure and its culture imply that it is hard to distinguish single actors being in the lead. Integration is a more complex process and requires more dynamics, than the law ‘keep it simple, stupid’ suggests.

**Conclusions:**

In the Dutch context integration implies a permanent pursuit of aligning mechanisms for integration. Sustainable integration requires long-standing efforts of all relevant stakeholders and cannot be achieved quickly. It may take a decade of consistently applying a mix of policy instruments.

## Introduction

In the Netherlands, there have been three major reforms to optimize the system of health, social and long-term care in the past fifteen years [1,2,3,4,5]. First, the healthcare reform in 2006 focused on acute healthcare, in the context of a public insurance system [3,4]. It intended to implement controlled market mechanisms by introducing competition and incentives for efficiency. Healthcare providers within the acute sector (GPs, hospitals, physiotherapists etc.) were expected to become more efficient, innovative, provide better quality of care and become more patient oriented. High-performing providers would be getting contracts, poor performers would lose their contracts. Healthcare insurers and care providers were expected to negotiate about quality and price. For patients there would be a mandatory (except for primary care) deductible to make them more aware of expenses and to reduce usage of services [3]. As such, it can be seen as a single sector reform focusing on the entire population in need of acute care.

Second, the long-term care (LTC) reform in 2015 has split up LTC into the new Long-term Care Act under the control of the regional care offices [5], the already existing Social Support Act by the municipalities [6] and the Health Insurance Act by insurance companies. It intended to realize a societal reorientation on formal and informal care, a shift from residential to non-residential care, supporting people to live independently, making better connections to social care, housing, employment and reducing the until then rapidly increasing long-term care expenditures [2]. This reform also implied a substantial repositioning of community-based long-term care to municipalities. District/community nursing was transferred to the Health Insurance Act, in order to better align with primary care [7]. As such, it was a reform across sectors and across governmental layers.

Third, in 2015 a large decentralisation also took place in youth care, transferring responsibilities from provinces, healthcare insurers and care offices to the municipalities [8]. The intention of this reform was to simplify the system of youth care and to make it more effective and efficient. Moreover, it intended to strengthen the resilience of youngsters and their families [8]. As such it was also a cross-sector reform across multiple governance levels focusing on a (broadly defined) target group in society.

All these reforms intended to create a more coherent and efficient care system, with new challenges for integration within and across sectors and governance levels. One of the core elements was decentralisation to local or regional authorities, with the idea that they are more capable to finetune care provision towards the needs of the population in their own specific context. With the reforms also came budgetary measures and new forms of contracting and financing.

Several issues of integration appeared to be challenging, e.g. the shift from institutional to home care, providing tailored and continuous care and support, reducing system complexity for citizens/service users and professionals, coordination between different disciplines and organizations, and between professionals and informal carers and citizens, information sharing (including the implementation of integrated client records), awareness of citizens and care professionals of appropriate services (including services that intent to assist people to navigate through the system), proactive case finding and case management for groups with long-standing multiple and complex needs, coordination of generic and specialised services, implementation and applying appropriate knowledge for new tasks at the various governmental layers [2,5,8,9].

To materialise these reforms, the Dutch government initiated a number of innovation and improvement strategies, applying a mix of instruments, for a range of stakeholders (such as care providers, professionals, healthcare insurers, municipalities and patients’ organizations), including many experiments and research projects. It intended to achieve a shift ‘from systems to people’ aiming at more coherence in the system and daily practice [10]. Moreover, the government intended to combat the gaps in the system that led to inappropriate and inefficient delivery of care and services [1].

In this paper, we will analyse five specifically selected integration strategies and their impact. The aim of our paper is to explore how the Dutch government took up responsibility across and within sectors at various levels of governance. We will analyse the strategies within the framework of the Laws of Integration as formulated by Walter Leutz [11,12,13]. First, we outline the key characteristics of the Dutch system.

## Policy context

The Euro Health Consumer Index places the Netherlands in the top three of European health systems, with high rankings on patients’ rights and information, accessibility, outcomes, range and reach of services and pharmaceuticals [14,15]. The Netherlands is corporatist in all its veins and has a highly diversified health and social care system. Care providers are organised in one or more umbrella organizations at national level. Professionals are also strongly organised according to their profession. Patients and clients are organised in both generic organizations, advocating the interests of general healthcare users, and categorical organizations, for patients with specific conditions [16].

Because of increasingly complex and longstanding health and social challenges in youth, older people, people with long-term mental health conditions, chronic diseases and disabilities, as well as a trend towards person-centred and holistic care, the need for integrative approaches is increasing [16]. A huge challenge is that the Dutch long-term care sector is one of the most expensive in the world [14].

The central government carries final responsibility for health and wellbeing of its citizens, holding ‘system responsibility’ to align measures and stakeholders [17]. Most of the services are run by independent public not-for-profit organizations as well as self-employed professionals, often collaborating in networks. A number of services are insurance-funded, a number merely tax-funded and quite a number of services are based on a combination of funding, including some out-of-pocket payments from users themselves [18]. Healthcare insurers (59 labels from 21 insurers in 11 financial conglomerates [19]) play a prominent role in acute healthcare and in long-term care (non-competitive, in 32 regional care offices). 355 municipalities are responsible for social care and youth care.

The central government has a role as integrator of legislation, funding and stakeholders to optimize health in the population. It has to ensure that all parties appropriately fulfil their distinct roles and responsibilities in the system. Amongst these roles and responsibilities are (without pursuing completeness) supervision and monitoring quality (Health and Youth Care Inspectorate), setting tariffs (National Healthcare Authority), determining what should be covered by public insurance at what quality requirements should be in place (National Healthcare Institute), eligibility testing for long-term care (Centre for Needs Assessment), collecting personal payments (Central Administration Office), and reimbursement of personal budgets (Social Insurance Bank) [16,18].

## Analytical framework and methods

In the next sections we will analyse five innovation and improvement programs that were initiated or facilitated by the government, and that focused on specific target groups, populations and/or categories of services. To analyse these programs, we used Walter Leutz’ Laws of Integration (including his amendments after the initial publication) as a theoretical and analytical framework [11,12,13] (see ***Box 1***).

**Box 1 Leutz’ Laws of Integration.**

1. You can integrate some of the services for all of the people, or all of the services for some of the people, but you can’t integrate all the services for all of the people.
2. Integration costs before it pays.
3. Your integration is my fragmentation.
4. You can’t integrate a square peg and a round hole.
5. The one who integrates calls the tune.
6. All integration is local.
7. Keep it simple, stupid.
8. Don’t try to integrate everything.
9. Integration isn’t built in a day.

From that, we derived twelve questions to analyse the programs (see ***Box 2***). We added the last question, as research accompanied the programs to evaluate their progress and results.

**Box 2 Format for program description: twelve questions (between brackets we refer to the particular law of Leutz).**

1. Background.
2. Who was the integrating actor? (5)
3. Who were the stakeholders involved? (2, 3 and 5)
4. Which stakeholders invested in the strategy? (2)
5. What was the estimated return on investment? (2)
6. Which populations were included, and which were excluded? (1, 4, 9)
7. What were the unresolved bottlenecks? (4)
8. Who was in the lead in the implementation? (5)
9. How was the strategy implemented at local level? (6)
10. How long did the development and implementation process take? (9)
11. What appeared to be too complex? (4, 7)
12. What was the role of (applied) science?

We selected five programs, which met the following criteria:

– Having a national scope;
– Addressing integration within a single sector and/or across sectors;
– Addressing integration across governmental layers;
– Being prepared and implemented over approximately the past decade;
– Program descriptions and evaluation studies being available.

More programs could have been chosen. For reasons of conciseness a selection was made of programs that addressed health and social care issues across the life span as well as a subpopulation as a whole, being target groups of the above-described reforms.

The following program descriptions are based on reports and official documents as well as peer reviewed publications. Each description was carried out by one of the authors and double-checked by a second author.

## Five integrative national programs

### Disease management

1. Background

In 2008, the Minister of Health, Welfare and Sport presented the programmatic approach for disease management for patients with chronic diseases. The aim was to stimulate collaboration between healthcare providers, by implementing so-called ‘care standards’. These care standards do not include an exhaustive, detailed description of what optimum care should comprise or by whom it should be delivered, but describe the desired organization and the quality requirements of functions and services, based on guidelines and practice-based evidence [20]. They also describe indicators for measuring, monitoring and improving the quality of care provided. The program on disease management also included integrated bundled payments, in which one organizational entity (called a ‘care group’) is contracted by the healthcare insurers to provide an integrated set of services as described in the care standards [21,22,23].

2. Integrating actor

The Ministry of Health, Welfare and Sport (further: the Ministry) described the program. The healthcare insurers contracted integrated service provision. General practitioners organised themselves in care groups that provide multidisciplinary care in a specific region.

3. Stakeholders

The National Health Authority and the Ministry established what integrated financing should entail [22]. An active role for patients, healthcare providers and healthcare insurers was expected by the Ministry [20].

4. Investors

The Ministry drafted a funding plan. The Netherlands Organization for Health Research and Development (ZonMw) commissioned the pilots and research, healthcare insurers funded actual care delivery [20,24].

5. Estimated return on investment

Systematic reviews showed contradictory results regarding the cost-effectiveness of this type of integrated care. In 2011, it was not yet possible to estimate whether disease management was saving costs. Results of cost-effectiveness evaluations at that stage proved to be ambiguous [21].

6. Included populations

Patients suffering from COPD/Asthma, Diabetes Mellitus type II, or cardiovascular disease.

7. Unresolved bottlenecks

Some essential aspects of integrated care, such as prevention, diagnostics, medicines, physiotherapy, nursing, and aids were not included in the integrated financing scheme. Instead, these were financed through other funding and out-of-pocket payments.

8. In the lead

The development of care standards is the joint responsibility of healthcare providers/professional associations, healthcare insurers and patients’ interest organizations. Regional agreements on integrated financing were the responsibility of the care groups and healthcare insurers.

9. Implementation strategy at local level

Care standards and integrated funding models gave impetus to set up regional care groups. They translated the national care standards into regionally disease-specific care programs. On the basis of that, care groups made regional agreements to provide tailored multi-disciplinary care, sometimes sub-contracting other partners.

At present 113 care groups participate in these disease management programs. Annual benchmarks are available showing improvements, underperformance, and practice variation [23].

10. Duration of development and implementation process

From the first projects with patients with diabetes to the implementation of care standards for other categories of patients the piloting period was two years (2008–2010). At present, integrated care for the particular categories of chronic patients covers 88% of the Dutch population [23].

11. Complexities

Mono-disease management programs are less suited for patients with multimorbidity. The programs only apply to a limited number of chronic diseases. Producing more care standards would cause more fragmentation of care delivery. Freedom of choice of patients is hindered by the mechanism of regional care groups, although patients do have the ability to choose other care providers.

12. Applied research

In 2010, an evaluation of this program was published which was used to further shape the national disease management program. The development of healthcare standards was recommended and a number of barriers in the funding system as well as in information technology were to be resolved. Also limitations in choice for patients, the need for clear responsibilities and accountabilities, governance of services providers and the too strong negation position of care groups were addressed [24]. The evaluation set the tone for future policies for the further development of disease management.

### Maternity care

1. Background

A decade ago, the Netherlands was facing too high perinatal mortality. In 2011, the Committee on Maternity Care (CMC) was established, comprising all stakeholders (care providers, patients organizations and healthcare insurers). It initiated the care standard ‘Integrated maternity care’, describing the characteristics and requirements of integrated care from preconception until six weeks after birth. Low-risk women usually give birth at home supported by midwives and, if needed, general practitioners. In the Dutch context this is considered a safe option and aligns with predominant cultural values about families and home life. Birth care is facilitated by the primary, secondary, and tertiary maternity care system. Primary care is facilitated by well-trained midwives working in the community, relatively short distances to hospitals and well organised ambulance services [25]. Secondary care is delivered by obstetricians and clinical midwives in general hospitals, tertiary care by obstetricians and clinical midwives in academic hospitals [26]. Important is the division between the (historically and characteristically feminine) midwives that focus on not merely physical aspects but also on the psychosocial aspects of pregnancy and birth, and (historically and characteristically masculine) obstetricians that focus on technical, interventionalist solutions to minimise risk of maternal and perinatal mortality [27].

To tackle high perinatal mortality, the CMC urged maternity care providers to increase collaboration through Maternity Partnerships (MPs), in which primary and secondary maternity care providing disciplines collaborate and work on quality improvement [28]. Later on, the Dutch government enabled pilots with bundled payments, whereby these partnerships would make agreements and contracts with healthcare insurers on integrated care combining different disciplines (gynaecology, maternity care, obstetrics, midwives). These organizations were coined ‘integrated maternity care organizations (IMCOs) [29]. The Dutch Healthcare Authority has recommended making bundled payment common practice after the pilots [29].

2. Integrating actor

Healthcare providers collaborate through IMCOs that are contracted by healthcare insurers. In MPs, maternity care providers collaborate and work on quality improvement without bundled payments [28,30].

3. Stakeholders

All parties involved in perinatal care are represented in the CMC. The CMC initiated the establishment of the integrated care standard for maternity care.

4. Investors

The establishment of IMCOs is accompanied by additional costing and reimbursement mechanisms. According to the contracts between IMCOs and healthcare insurers, these costs are (partially) reimbursed. Furthermore, IMCOs can obtain financial support by the CMC, the Ministry, or the Taskforce Maternity Care.

5. Return on investment

The first results of an evaluation demonstrated a small but opposite financial effect, in favour of the IMCOs. There was not yet a clear effect on the quality of care [29,30,31]. A recent evaluation stated it was difficult to calculate the return of investment of collaboration and network development, since MPs and IMCOs rarely monitor their collective goals.

6. Included populations

Women requiring maternal care were included in the experiment.

7. Unresolved bottlenecks

So-called ‘bundle-breakers’ may occur when both a monodisciplinary as well as an integrated service is declared. This is prohibited according to current funding regulations. Bundle-breakers are caused by information-asymmetry between healthcare insurer and provider: the insurer has the information on declarations of non-IMCO providers, the IMCO does not. Further, timely exchange of financial information between providers and insurers is not yet possible in all IMCOs.

8. In the lead

The Ministry provided opportunities to experiment with integrated financing. The care providers, healthcare insurers and patients’ organizations at national level joined up and developed the care standard and the organizations models. The National Health Authority developed and evaluated the bundled payments methodology [32].

9. Implementation strategy at local level

The care standard was implemented according to a plan, established by the CMC. The IMCOs and healthcare insurers made financial agreements about aspects quality of care, overhead costs, and accountability.

10. Duration of development and implementation process

The government announced the first additional measures to combat perinatal mortality in 2008. The CMC was established in 2011. The care standard for integrated perinatal care was presented in 2016 with an implementation phase of four years. The pilot of integrated financing of perinatal care started in 2017 and lasts until 2021.

11. Complexities

To decrease the administrative burden of IMCOs, healthcare insurers signed a voluntary agreement to follow the same general rules. Nevertheless, IMCOs experience a large amount of bureaucracy because the various healthcare insurers apply different contract agreements. An evaluation in 2021 found that IMCOs and MPs face several barriers, dependent on the phase of collaboration, from the start of practical collaboration to functioning as a fully developed, integrated network or as one organization. In the first phase, the barriers mostly exist on micro level, between individual care providers or practices, while the barriers on macro (or national) level become more predominant as the collaboration matures. According to the researchers, the evaluation shows that various MPs and IMCOs handle these barriers differently, and that, in theory, most barriers can be circumvented through interventions or practical solutions.

12. Applied research

The pilot with bundled payments for IMCOs has been evaluated by investigating measures such as costs and quality, as well as the experiences of healthcare insurers and IMCOs [29,31,32].

This evaluation was used by the Dutch Healthcare Authority to state that bundled payment should be further implemented in maternity care. Furthermore, an evaluation was presented in 2021 on the experiences of MPs and IMCOs of the development of collaboration and potential barriers.

### Youth Care Act

1. Background

In 2015, the new Youth Care Act was put into place to provide more timely, integrated and cohesive care in local teams. Three years after implementation an evaluation has shown that these goals had not yet been achieved [8]. As a response to this evaluation, the program ‘Care for the youth’ commenced in 2018, starting collaboration between the government, municipalities, client organizations and care providers to achieve better care and support for children and people in their environment [33]. The new act has six goals: better access to youth support; more children growing up at home; giving all children the chance to develop as much as possible; assisting vulnerable young adults in becoming independent; better protection for young adults when their development is endangered; investing in craftmanship of youth care professionals and establishing a healthy work environment.

2. Integrating partner

In 42 ‘youth regions’, municipalities and youth support providers collaborate to transform youth care into local teams of professionals.

3. Stakeholders

The central and local governments, care and support providers and client organizations.

4. Investors

The expenses for the program are divided 50/50 between the municipalities and the Ministry.

5. Return on investment

In 2020, an evaluation describes that some steps have been taken concerning the transformation goals but that there is still a long way to go [34]. For example, when comparing the numbers of 2015 to the ones of 2020, more youngsters and families experience that they are involved in decision making (87% vs 78%). However, the proportion of youngsters and families that think they received help quickly remained approximately the same (64% vs. 61%).

6. Included populations

Children, young adults, and families.

7. Unresolved bottlenecks

In aspects of youth support that touch upon other domains (e.g., housing, education), no positive results have been found yet. Furthermore, within youth care issues such as collaboration, expertise and access still are open for improvement [34].

8. In the lead

All stakeholders collaborate in partnerships. The youth regions are responsible for the eventual transformation of youth care in their region.

9. Implementation strategy at local level

The program goals were supposed to be achieved by the municipalities in the youth regions. They could use a transformation budget to close ‘regional youth deals’. Support teams consisting of both regional and national ambassadors assist and advice municipalities in the transformation process.

10. Duration of development and implementation process

In 2018, the program actions were established through multiple client representatives and organizations that work with and for youths. The program is due to last until 2021. However, the evaluation in 2020 advised to extend the program since the transformation process is complex and slower than foreseen [34]. The stakeholders set up a so-called ‘learning environment’ surrounding children and families.

11. Complexities

The program aimed at solving the issues of all children locally, by professionals in local teams and care institutions (with some exceptions where children are referred to regional expert teams). The evaluation showed that some families did not receive the necessary care, even though this care is available locally. Furthermore, appropriate solutions are yet not available for families with very complex needs [34].

12. Applied research

A knowledge development program ‘What works in youth care’ was set up by ZonMw, academic workplaces ‘transformation youth’ were involved to develop knowledge and skills, and national knowledge institutes (e.g., Movisie, Netherlands Youth Institute) were involved for providing applicable information.

### The National Care for Older People Program (NPO)

1. Background

The National Care for Older People Program was an initiative to develop and collect knowledge on frail older people, to assess their needs and provide better-tailored integrated and person-centred care, based on scientific evidence. The aim was to improve independence, preservation of functions, and, as a consequence, less use of care and treatment [35,36]. The program commenced in 2008 and consisted of three steps: the formation of regional geriatric care networks, delivering innovative and transition projects, and nationwide dissemination and implementation of effective project interventions and results.

2. Integrating partner

The program involved + 650 parties in eight different regional geriatric care networks around eight University Medical Centres (UMCs).

3. Stakeholders

Stakeholders in the networks included general practitioners, municipalities, home care organizations, pharmacies, welfare organizations, organizations of older people, nursing homes, hospitals, healthcare insurers, universities, and education institutes. ZonMw worked in close collaboration with the Dutch Federation of University Medical Centres, the eight UMCs and with older people through the Central Collaborating Elderly Organizations. Furthermore, knowledge organizations, such as Vilans and Movisie were involved for knowledge dissemination and utilization.

4. Investors

The ZonMw program had a budget of 88 million Euros, provided by the Ministry of Health Welfare and Sport [35]. ZonMw funded the various regional networks and projects, as well as research.

5. Return on investment

The output of the NPO was diverse, including scientific, participative (participation of older people in the projects), collaborative, and practical yields.

6. Included populations

All NPO projects together included + 43.000 (frail) older individuals in the population.

7. Unresolved bottlenecks

During the project, decentralisation reforms were announced in long-term and social care (see Introduction), leading to shifting priorities in the sector instead of working on NPO activities. Also, mainly older people of higher social and cultural ‘capital’ and vitality actively participated in the projects, not the target group. In practice, the scale of the NPO networks did not correspond with the locally needed networks for collaboration in care for older people.

8. In the lead

ZonMw was in the lead for the whole NPO program and the eight UMCs for the regional networks [36].

9. Implementation strategy at local level

All networks worked according to their grant application to ZonMw, including administrative agreements on the regional organization of care and support for older people with complex needs. Furthermore, the networks had to include as many as possible stakeholders. The implementation goal was to deliver full regional coverage. The UMCs each got a yearly budget of 200.000 Euros to cover coordination activities.

10. Duration of development and implementation process

Starting in 2008, the program aimed to realize its goals within four years. This was too ambitious, and the program was extended with another four years. During the program, the focus shifted from empirical research towards reforming the local care system, based on what older individuals themselves found important, and the focus on medical care broadened to social care for older people.

11. Complexities

The implementation and dissemination of successful experiments appeared to be complex, taking a lot of time and efforts [37]. After the program and its funding ended, only a few networks continued, within their own funding structures.

12. Applied research

The program was very much research-led by the UMCs. It resulted in many scientific publications, PhD theses and practical guidelines. A database was established that contains the research data of + 43.000 older individuals and + 9.000 informal caregivers. On the other hand, innovations in daily practice were hard to implement on a sustainable basis [36].

### Dementia Care Program

1. Background

In 2011, ‘Deltaplan Dementie’ was initiated as the national strategy to improve the life of people with dementia, through collaboration between the government, societal parties, researchers and business work [38]. The Deltaplan focused on three pillars: research to prevent and cure dementia [39], improving dementia care, and establishing a dementia-friendly society. The pillar ‘improving dementia care’ contains a 4-year Dementia Care Program, which was an improvement program, providing advice on how to organize, finance and improve integrated dementia care, sharing knowledge on care for people with dementia, the establishment of a dementia register and the publication of a new care standard for dementia care [40].

2. Integrating partner

Focus of the Program is on building regional infrastructures of dementia care networks, containing all relevant stakeholders in the field. In total there are + 65 dementia care networks, on the level of large cities, regions or provinces.

3. Stakeholders

The Program was a collaborative of five knowledge institutes: Movisie, Nivel, Pharos, Trimbos-institute and Vilans.

4. Investors

The program was funded by the Ministry, that also funded the research pillar of the Deltaplan (Memorabel) [39].

5. Return on investment

According to an evaluation of the whole Deltaplan (including the improvement pillar), stakeholders state that the program has helped to get a clear view on the issues in care practice surrounding dementia [41].

6. Included populations

People with dementia and their informal caregivers during the whole ‘patient journey’.

7. Unresolved bottlenecks

The inclusion of all regional partners, the multidomain coverage and the implementation power of the differently structured dementia care networks varies widely, also because of lack of formal authority and (structural) funding of the networks. This was one of the triggers for the Ministry to launch a national dementia strategy 2021–2030 that also challenges the dementia care networks to take the lead in full implementation of the national care standard by 2025 [42]. However, regular funding for networks or their coordination is not arranged yet. A new research program as a follow-up of Memorabel will start in 2021, with a accumulating investment of 148 million Euros until 2030.

8. In the lead

The collaborative of Vilans, Movisie, Nivel, Pharos, Trimbos-institute ran the improvement program, in close collaboration with the field, patient organization Alzheimer Netherlands and the Ministry.

9. Implementation strategy at local level

The dementia care network and regional integrated care networks could request support whenever they faced problems or wanted to take steps for further development. This included advise, support for the implementation processes, and tools for professionals. Further, the program provided consultants who worked together with dementia care networks on issues that are prevalent in more than one region (for instance young persons with dementia). Many dementia care networks developed regional action plans. Health care insurers required such plans to consider funding of the networks. Most of them did not live up to this promise.

10. Duration of development and implementation process

The Dementia Care Program ran from 2017 until mid-2021. Some elements of the program will continue as part of the new national dementia strategy 2021–2030, e.g., the Dementia Register and the implementation of the dementia care standard in all regional dementia care networks [42].

11. Complexities

In 2016, stakeholders came together to improve case management for people with dementia. An action plan was launched, but was difficult to fully realise because of varying opinions in the field. Finally, it became part of the care standard for dementia. The latter has been developed by 22 national parties of patients, healthcare providers, professionals’ organization in various domains, healthcare insurers, and was authorized by the National Healthcare Institute in April 2020 [43].

12. Role of applied research

Prior to multiple initiatives, pilot implementation trajectories were conducted, including a light procedure of monitoring goal attainment scores. Furthermore, the spread of knowledge products and research output is enabled by the infrastructure of the Deltaplan. The improvement program provides a network to disseminate results. Also, a register was developed, to support clinical practice and future research.

## Discussion

In this section we discuss how the Dutch government took up its system-responsibility for implementing integrated care programs in the last decade. Thereafter, we will discuss how Leutz’ Laws of Integration were applied as an analytical framework for researching the past decade’s programs.

The strategies and policy instruments the Dutch government used to reform sectors and to integrate services and systems took place within different changing legislative contexts and with a focus on various target groups. Core ingredients of the programs were care standards, funding instruments, local/regional structures, applied research, pilots and implementation programs. In single sector reform (4.1 and 4.2) quality standards, commissioning requirements and responsibilities were more clear cut, with healthcare insurers and local collaborative care providers having a key role in sectoral integration [44]. The across sector and across governmental layer programs (4.3, 4.4 and 4.5) aiming at whole system integration were less clearly guided by care standards and funding instruments, but were of a more bottom-up approach.

In spite of significant system reforms and large implementation programs many integration issues remain unresolved. As such the frequently noted need for governance and accountability as requirements for well-integrated care appears to be difficult to achieve in the Dutch context [45]. The system is a mixture of a Bismarckian and a Beveridge system, with elements of controlled competition within a welfare state model that intends citizens to take up their own responsibility. Competition between healthcare insurers and providers, as well as barriers between governmental layers, new tasks for municipalities, different legislation and barriers within acts are still hampering integration [46].

Dutch policies focused on selectively integrating parts of the system, not aiming at whole system integration. In the programs there was a mix of the type of integration, the level at which integration took place, the process, the breadth and the degree of integration [44]. Even within single sector programs barriers appeared to exist, hindering horizontal and vertical integration [44,47]. The different reimbursement schemes and quality standards contain unsurmountable barriers.

On the other hand, in particular in the single sector programs progress has been made. The implicit method of across sector integration was to decentralise responsibilities and initiate pilots, accompanied by evaluative research. In fact, this can be seen as way to establish goal-directed networks, defined as ‘an intentional, cross-organizational integration in function of a well-defined, common target’, with a ‘certain degree of stability in terms of mission, composition, collaboration and governance’ [48]. It appears that – in terms of Goodwin – people-centred integration and whole-system integration for particular groups with multiple long-lasting needs cannot be resolved by merely reforming legislation or by launching an improvement program [44]. Horizontal and vertical integration with multiple actors is feasible with the right support of instruments like care (or quality) standards, funding mechanisms such as bundled payments, and regional or local governance structures. But it always takes quite some time.

Although national program and policy measures were issued, the speed of implementation and the degree of integration differs significantly across the country [44]. This demonstrates that factors such as leadership, organizational cultures and the social dimensions make the difference [47,49]. The position of patients, clients or services users was most prominent in the National Care for Older People Program (4.4), but as such, it is not a guarantee for success.

As mentioned above, in this paper we used the Laws for Integration as formulated by Leutz ([11,12,13] as an analytical framework. The theorems of this framework were to a large extent confirmed in the five integrated care programs. The laws ‘You can integrate some of the services for all of the people, or all of the services for some of the people, but you can’t integrate all the services for all of the people’ and ‘don’t try to integrate everything’ were evident in the segmented strategy to issue programs and policy measures for specific target groups and not aiming at whole system integration. The law ‘Integration costs before it pays’ was clearly demonstrated, as was the statement ‘Integration isn’t built in a day’. It took at least a decade to implement integration at a large scale, and even then, significant shortcomings existed. The chronic disease management program for a limited number of patient categories was implemented for the vast majority of this population across the country. Integrated maternity care is literally and figuratively in its infant’s phase. It started later than chronic disease management but is now making progress. However, there is a large diversity. Integration of youth care is still work in progress. The care for older people program and the dementia care program are challenging in similar respects. Further, initial investments seemed to be required for, for instance, applied research and development, and extra overhead costs.

Leutz’s laws ‘Your integration is my fragmentation’ and ‘You can’t integrate a square peg and a round hole’ are implicit in all demonstrated programs. In all examples of reforms and programs, boundaries were defined to demarcate target groups, policy actors and legislative frameworks, to include some and to exclude others. This demonstrates that integration at one point does not cover all, or even can lead to more fragmentation elsewhere. A limited number of focal points for the various measures was defined to make a program feasible, leaving aside other related fields and issues.

The law ‘The one who integrates calls the tune’ is difficult to demonstrate in the Dutch context. Being a corporatist nation in all its nerves, integration appears to be a joint effort of a wide variety of system parties. There are certainly various power structures, but there is no single integrator. The statement ‘All integration is local’ is key in all programs, however, within a multi-layer structure of requirements and incentives. It depends on the choices for the level of governance and the commissioning actors where ultimate responsibilities lie. In all cases more levels of governance are active, depending on scale and the predominant legislative framework.

The rule ‘Keep it simple, stupid’ appears to be an unattainable goal, especially in the combined fields of health, long-term and social care. The interconnections between the various systems and societal impacts are too manifold to meet the often longstanding multiple needs of those who are dependent on integrated care. Organising integrated services is in all cases a wicked problem or challenge.

### Strengths and limitations

The present analysis addresses five integration strategies initiated or supported by central government in the Netherlands. We based ourselves on existing research and program descriptions. These programs were incorporated in a mix of other policy instruments and changing policy configurations, which have influenced the impact of the programs. Our analysis only addresses policies in the Dutch context of the past ten to fifteen years. Comparative research could further clarify the key mechanisms of integration at system level.

The programs that are analysed in this paper are chosen by the authors, based on the inclusion criteria, the authors’ long standing involvement and experience in the Dutch system and the availability of proper documentation. Choices to organise the vast information on the twelve categories of analyses is to some extent subjective, although all information is double checked by the authors.

The lessons from the present COVID-19 crisis are not incorporated in this analysis, but are based on the ‘old’ normal. They are probably relevant to design integration in the future. But as of yet, the ‘new’ normal is far from clear.

## Conclusions

The governmental programs to further integration differ in scope and impact for within sector and across sector and across government strategies. Key is the question: who is the integrator to whom means, powers and instruments are allocated to integrate service delivery at the right level [50]? This question not only requires a systemic response, but also needs to be addressed from a social behavioural perspective [49].

Information and registration were key features to be solved in all programs and in all integration efforts, although no law of Leutz was formulated for this aspect of integration.

Furthermore, the scale of services and governance appears to be a crucial factor in integration. Even when services and integration processes are decentralised there need to be mechanisms to scale up for more specialised expertise. All programs that we studied had their own local or regional implementation structures, none of them coinciding. Integration is a challenge on various levels, with various responsibilities and logistic but also social-relational requirements. It is important to rethink the micro, meso and macro challenges of scale [51].

Participation of citizens and patients was a challenge. In none of the integration efforts they were really in the lead. Research appeared to be supportive in developing and evaluating integrated care models, in addition to policy measures, funding, legislative measures and implementation strategies. According to our personal observations mutual learning between programs was limited, except for where personal unions existed. Within programs learning was well facilitated.

The impact of the Dutch corporatist system complexity on the speed and quality of integration is difficult to assess. Most of the problems envisaged are observed across systems [45,47,52]. Multi-nation comparative studies are to shed more light on this issue. However, the national and local cultural and behavioural characteristics should not be underestimated. Within one policy system, a wide variety of integration exists.

In any case, the corporatist Dutch system integration requires a permanent pursuit of aligning mechanisms for integration. The current COVID-19 crisis triggered a rapid speeding up of integration [53]. At this moment it is unsure whether this intensified integration is sustainable.

In sum, the overview of ten years of integration policies in the Netherlands shows that progress has been made, but also that system reforms and legislation cannot solve all problems. The complexity of health, social and long-term care will remain an immense challenge for integration within and between sectors and governmental layers.
